# Stratified care integrated with eHealth versus usual primary care physiotherapy in patients with neck and/or shoulder complaints: protocol for a cluster randomized controlled trial

**DOI:** 10.1186/s12891-021-03989-0

**Published:** 2021-02-05

**Authors:** Mark L. van Tilburg, Corelien J. J. Kloek, Martijn F. Pisters, J. Bart Staal, Johanna M. van Dongen, Marjolein de Weerd, Raymond W. J. G. Ostelo, Nadine E. Foster, Cindy Veenhof

**Affiliations:** 1 Expertise Center Healthy Urban Living, Research Group Innovation of Human Movement Care, HU University of Applied Sciences Utrecht, Heidelberglaan 7, 3584 CS Utrecht, the Netherlands; 2 Center for Physical Therapy Research and Innovation in Primary Care, Julius Health Care Centers, Utrecht, the Netherlands; 3 Department of Rehabilitation, Physiotherapy Science and Sports, UMC Utrecht Brain Center, University Medical Center Utrecht, Utrecht University, Utrecht, the Netherlands; 4 Research Group Empowering Healthy Behaviour, Department of Health Innovations and Technology, Fontys University of Applied Sciences, Eindhoven, the Netherlands; 5 Musculoskeletal Rehabilitation Research Group, HAN University of Applied Sciences, Radboud University Medical Centre, Nijmegen, the Netherlands; 6 Radboud Institute for Health Sciences, IQ Healthcare, Radboud University Medical Center, Nijmegen, the Netherlands; 7 Department of Health Sciences, Faculty of Science, VU University, Amsterdam Movement Sciences Research Institute, Amsterdam, the Netherlands; 8 Department of Epidemiology and Data Science, Amsterdam University Medical Center, Amsterdam Movement Sciences Research Institute, Amsterdam, the Netherlands; 9 Primary Care Centre Versus Arthritis, School of Medicine, Keele University, Keele, UK; 10 STARS Education and Research Alliance, School of Health and Rehabilitation Sciences, The University of Queensland, Brisbane, Australia

**Keywords:** Physiotherapy, Neck pain, Shoulder pain, Musculoskeletal disorders, Stratified care, Telemedicine, eHealth, Blended care

## Abstract

**Background:**

Neck and shoulder complaints are common in primary care physiotherapy. These patients experience pain and disability, resulting in high societal costs due to, for example, healthcare use and work absence. Content and intensity of physiotherapy care can be matched to a patient’s risk of persistent disabling pain. Mode of care delivery can be matched to the patient’s suitability for blended care (integrating eHealth with physiotherapy sessions). It is hypothesized that combining these two approaches to stratified care (referred to from this point as Stratified Blended Approach) will improve the effectiveness and cost-effectiveness of physiotherapy for patients with neck and/or shoulder complaints compared to usual physiotherapy.

**Methods:**

This paper presents the protocol of a multicenter, pragmatic, two-arm, parallel-group, cluster randomized controlled trial. A total of 92 physiotherapists will be recruited from Dutch primary care physiotherapy practices. Physiotherapy practices will be randomized to the Stratified Blended Approach arm or usual physiotherapy arm by a computer-generated random sequence table using SPSS (1:1 allocation). Number of physiotherapists (1 or > 1) will be used as a stratification variable. A total of 238 adults consulting with neck and/or shoulder complaints will be recruited to the trial by the physiotherapy practices. In the Stratified Blended Approach arm, physiotherapists will match I) the content and intensity of physiotherapy care to the patient’s risk of persistent disabling pain, categorized as low, medium or high (using the Keele STarT MSK Tool) and II) the mode of care delivery to the patient’s suitability and willingness to receive blended care. The control arm will receive physiotherapy as usual. Neither physiotherapists nor patients in the control arm will be informed about the Stratified Blended Approach arm. The primary outcome is region-specific pain and disability (combined score of Shoulder Pain and Disability Index & Neck Pain and Disability Scale) over 9 months. Effectiveness will be compared using linear mixed models. An economic evaluation will be performed from the societal and healthcare perspective.

**Discussion:**

The trial will be the first to provide evidence on the effectiveness and cost-effectiveness of the Stratified Blended Approach compared with usual physiotherapy in patients with neck and/or shoulder complaints.

**Trial registration:**

Netherlands Trial Register: NL8249. Officially registered since 27 December 2019. Date of first enrollment: 30 September 2020. Study status: ongoing, data collection.

## Background

Worldwide, 1.3 billion people are affected by musculoskeletal (MSK) conditions each year [1]. Two common MSK presentations are neck and shoulder complaints [1–4]. Patients with neck and/or shoulder complaints experience pain and disability, resulting in high societal costs due to e.g. healthcare usage, work absenteeism and presenteeism [5]. In the Netherlands, neck and/or shoulder complaints are predominately managed in primary care by physiotherapists. The latest clinical guidelines recommend physiotherapists provide patient-centered care, assess psychosocial factors, educate patients by providing them with information about their condition and self-management options, and provide treatment that addresses physical activity and exercise [6–11]. However, like other musculoskeletal conditions, there is no ‘one size fits all’ strategy to manage patients with neck and/or shoulder complaints [6]. Stratified care is a model of care with two components; firstly the use of a tool to identify subgroups of patients and then matching treatments to patients in each subgroup [12]. In this study, two approaches to stratified care (referred to from this point as Stratified Blended Approach) are combined to match subgroups of patients with neck and/or shoulder complaints to the most appropriate content and intensity of physiotherapy and mode of care delivery.

Content and intensity of physiotherapy can be matched to a patient’s risk of persistent disabling pain, using prognostic stratification. Among patients with neck and shoulder complaints, the Keele STarT MSK Tool can be used to classify patients as either having a low, medium, or high risk of developing persistent disabling pain [13, 14]. The Keele STarT MSK Tool contains ten items assessing a patient’s function and disability, pain and coping, comorbidity and the impact of pain. The tool has shown good predictive and discriminative ability among UK primary care patients at low, medium and high risk of persistent disabling pain [15]; (Dunn K, Campbell P, Lewis M, Hill J, van der Windt D, Afolabi E, et al: Refinement and validation of the Keele STarT MSK Tool for stratifying patients with musculoskeletal pain, submitted). Additionally, the Dutch version of the Keele STarT MSK Tool showed sufficient to good validity and reliability among Dutch primary care patients with musculoskeletal pain (van den Broek A, Kloek C, Pisters M, Veenhof C: Validity and reliability of the Dutch STarT MSK tool in patients with musculoskeletal pain in primary care physiotherapy, submitted). The information about patient subgroups can be used to match patients to recommended treatment options. Suitable matched treatment options for patients with neck and/or shoulder complaints in the Dutch health system were previously determined in a development and feasibility study (van Tilburg M, Kloek CJJ, Pisters MF, Staal JB, Ostelo WJG, Foster NE, et al: Development & feasibility of a stratified approach integrated with eHealth in patients with neck and/or shoulder complaints, in preparation). Content and intensity of physiotherapy for patients at low risk is suggested to focus on reassurance, information on neck/shoulder complaints, personal etiology, self-management options, and the importance of adequate physical activity/exercise behavior over 3–4 sessions, on average. Recommended treatment options for patients at medium risk are similar to low risk and the physiotherapist should additionally consider providing passive or active joint mobilization techniques, in combination with functional exercise therapy over 6–9 sessions, on average. Physiotherapy for patients at high risk should additionally focus on addressing patient’s specific physical and psychosocial obstacles to recovery, using a combination of physical and psychological approaches, including pain education over 8–12 sessions, on average (van Tilburg M, Kloek CJJ, Pisters MF, Staal JB, Ostelo WJG, Foster NE, et al: Development & feasibility of a stratified approach integrated with eHealth in patients with neck and/or shoulder complaints, in preparation).

Mode of care delivery can be matched to the patient’s suitability for integrating eHealth with physiotherapy sessions, called blended care [16]. In order to determine whether a patient is suitable to receive a blended physiotherapy intervention, the Dutch Blended Physiotherapy Checklist was recently developed [16]. Items of this checklist assess the patients’ motivation, safety, equipment, digital skills, health literacy, self-management, time, and financial situation [16]. Recent studies showed the potential of a blended care delivery mode, in which physiotherapy sessions are integrated with a smartphone application to stimulate patients’ ability to manage their musculoskeletal problems independently, outside of treatment sessions [17–19]. An example of a blended physiotherapy intervention is e-Exercise, which has been developed for patients with hip and knee osteoarthritis, low back pain, and recently for neck and/or shoulder complaints (van Tilburg M, Kloek CJJ, Pisters MF, Staal JB, Ostelo WJG, Foster NE, et al: Development & feasibility of a stratified approach integrated with eHealth in patients with neck and/or shoulder complaints, in preparation); [17–21]. E-Exercise is an integration of physiotherapy sessions with a smartphone application consisting of an information module, an exercise module, and a physical activity module. The e-Exercise functionalities of these three integrated modules differ per risk profile (low, medium or high risk of persistent disabling pain) and will be personalized for each individual patient. In the smartphone application, several behavior change techniques are used to support self-management skills and improve adherence to exercise and physical activity recommendations. Examples of such behavior change techniques are goal setting, assignments, tailored feedback, self-monitoring, visualization of treatment progress and content matching [22]. These behavior change techniques were found to enhance healthy behavior, such that recurrences of symptoms might be prevented, which might in turn lead to a reduction in healthcare and societal costs [21, 23]. However, blended care may not be suitable for every patient and is not expected to be effective in this subgroup [16]. Therefore, a paper-based workbook, with similar content to e-Exercise, was developed for this subgroup of patients (van Tilburg M, Kloek CJJ, Pisters MF, Staal JB, Ostelo WJG, Foster NE, et al: Development & feasibility of a stratified approach integrated with eHealth in patients with neck and/or shoulder complaints, in preparation).

The Stratified Blended Approach is a model of care that can assist physiotherapists in deciding the content and intensity as well as mode of care delivery of primary care physiotherapy (van Tilburg M, Kloek CJJ, Pisters MF, Staal JB, Ostelo WJG, Foster NE, et al: Development & feasibility of a stratified approach integrated with eHealth in patients with neck and/or shoulder complaints, in preparation). Stratified care has the potential to optimize treatment benefits and increase the efficiency of healthcare, because patients are more likely to receive treatments that meet their needs and less likely to receive unnecessary treatments [24, 25]. In the literature, we see a growing amount of evidence for risk stratification on the one hand and integrating technology on the other hand. Integrating knowledge from both of these fields might improve the effectiveness and cost-effectiveness of physiotherapy care for patients with neck and shoulder complaints. This paper describes the protocol for a cluster randomized controlled trial to determine the clinical and cost-effectiveness of the Stratified Blended Approach compared to usual physiotherapy. A cluster design will be used to avoid the risk of contamination between the Stratified Blended Approach and usual physiotherapy.

- Our primary research aim is to investigate the clinical effectiveness of the Stratified Blended Approach for patients with neck and/or shoulder complaints on pain and disability over 9 months, compared to usual physiotherapy care.

Our secondary aims are twofold:

- to investigate the effectiveness of the Stratified Blended Approach for patients with neck and/or shoulder complaints on pain intensity, health-related quality of life, illness perceptions, self-management skills, physical activity, exercise adherence, self-perceived effect and satisfaction at 3 and 9 months, compared to usual physiotherapy care;
- to investigate the cost-effectiveness and cost-utility of the Stratified Blended Approach for patients with neck and/or shoulder complaints, compared to usual physiotherapy care.

## Methods

### Trial design and setting

**Fig. 1:**
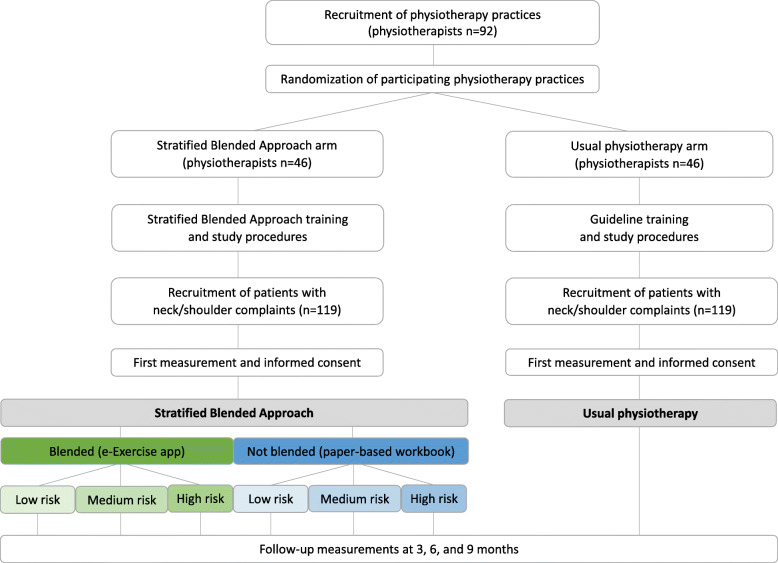
Flowchart of the trial design

A multicenter, pragmatic, two-arm, parallel-group, cluster randomized controlled trial (cRCT) will be conducted. This trial is approved by the Medical Ethics Committee Utrecht, the Netherlands, with number: NL69963.041.19. Physiotherapists will be recruited from primary care physiotherapy practices across all regions of the Netherlands. After recruitment, participating primary care physiotherapy practices will be randomized to either offer the Stratified Blended Approach or usual physiotherapy care by a computer-generated random sequence table generated using SPSS, using 1:1 allocation. The number of physiotherapists per participating practice (1 or > 1) will be used as a stratification variable. Although individual physiotherapists are identified as clusters, physiotherapy practices will be the unit of randomization in order to prevent contamination between physiotherapists. Due to the nature of the intervention, blinding of participating physiotherapists is not possible. However, neither physiotherapists nor patients in the usual physiotherapy care arm will be informed about the Stratified Blended Approach arm. A flowchart of the trial design is shown in Fig. 1.

### Participants

#### Physiotherapists

Patients will be identified, invited and recruited by Dutch primary care physiotherapy practices. We aim to recruit 92 physiotherapists. Physiotherapists are eligible to participate if they have at least four patients with neck and/or shoulder complaints applying to them for physiotherapy treatment each month. All physiotherapists, regardless of experience and education or specialization (e.g. manual therapy), are eligible to participate. Physiotherapists in the usual physiotherapy arm will receive half a day of training in relevant physiotherapy practice guidelines [9–11] and research procedures. Physiotherapists in the Stratified Blended Approach arm will be trained to deliver the Stratified Blended Approach, relevant guidelines [9–11], and research procedures during two training sessions, both of which will last half a day.

#### Patients

We aim to recruit a total of 238 patients. Recruitment of patients with neck and/or shoulder complaints will start in September 2020 after randomization of the physiotherapy practices and training of the participating physiotherapists. All patients of 18 years or older consulting for physiotherapy treatment for neck and/or shoulder complaints will be orally informed about the study and invited to participate in the data collection by the participating physiotherapy practice staff, during the initial registration. Every patient that is potentially eligible and is not invited by the physiotherapy practice staff during the initial registration, will be invited by participating physiotherapists. If the patient is willing to participate in the data collection, contact details will be sent to the researcher using a secured messenger service, called Siilo (www.siilo.com). Subsequently, the researcher will email an information letter to the patient, containing information about the purpose of the study, trial design, study procedures, potential risks and benefits of participation, expected duration of the study, confidentiality of personal identification and demographic data, so that it is clear that participation in the data collection is entirely voluntary. There are two different information letters. All are informed they are being invited to participate in a randomized trial. Patients in the Stratified Blended Approach arm will be informed about the content of the Stratified Blended Approach. Patients in the usual physiotherapy arm will not be informed about the content of the Stratified Blended Approach. After at least four hours, the researcher will contact the patient by phone and will check whether the patient read and understood the information letter. If so, the researcher performs an initial screening of the in- and exclusion criteria. Patients with sufficient mastery of the Dutch language are eligible for participation if they suffer from subacromial complaints, biceps tendinosis, shoulder instability or non-specific musculoskeletal complaints of the neck and/or shoulder (not caused by acute trauma (fracture or rupture) or by any systemic disease) [7, 26]. Patients will be excluded if there are neck and/or shoulder complaints caused by a specific pathology (e.g. shoulder pain with loss of active and passive range of motion [frozen shoulder], vertebral fracture, tendon rupture, Parkinson’s disease, herniated nucleus pulposus, cervical stenosis), except for subacromial impingement, biceps tendinosis and shoulder instability. After the researcher informed the participant about the study and assessed his or her eligibility, an informed consent form will be sent to the participant by mail and the first assessment will be sent by the researcher to the patient as soon as possible. After consenting to participate in the data collection, the patient is asked to send the signed informed consent form back to the research team by mail. An extra check of the in- and exclusion criteria will be performed by the physiotherapist during the first physiotherapy session. There will not be a maximum number of patients that can be included per physiotherapist. Concomitant interventions are permitted.

### Intervention

#### The stratified blended approach

The Stratified Blended Approach was developed in close collaboration with physiotherapists, patients, a commercial eHealth entrepreneur and health researchers, using the Center for eHealth Research (CeHRes) Roadmap (van Tilburg M, Kloek CJJ, Pisters MF, Staal JB, Ostelo WJG, Foster NE, et al: Development & feasibility of a stratified approach integrated with eHealth in patients with neck and/or shoulder complaints, in preparation); [27]. During the development process, the feasibility of the Stratified Blended Approach was evaluated in a feasibility study and several amendments were made where necessary (van Tilburg M, Kloek CJJ, Pisters MF, Staal JB, Ostelo WJG, Foster NE, et al: Development & feasibility of a stratified approach integrated with eHealth in patients with neck and/or shoulder complaints, in preparation).

In the Stratified Blended Approach arm, physiotherapists will use two stratification tools and two practical tools to match the content and intensity as well as mode of care delivery to the patient. These tools will be integrated with physiotherapy treatment sessions (either face-to-face or video consults) and are explained in detail in the following paragraphs [6–11]. First, physiotherapists will use the two stratification tools to decide the most suitable content and intensity as well as mode of care delivery of primary care physiotherapy. The content and intensity of physiotherapy will be matched to the patient’s risk of persistent disabling pain as assessed with the Keele STarT MSK tool (i.e. low, medium, or high risk). The mode of care delivery of physiotherapy will be matched to the patient’s suitability for blended care as assessed using the Dutch Blended Physiotherapy Checklist (i.e. yes or no). Thus, theoretically there are six matched treatment groups, see Fig. 1. Second, physiotherapists will receive two practical tools to provide the matched treatment of the mode of care delivery. If considered suitable for blended care, the patient will receive a blended physiotherapy treatment (e-Exercise), in which a smartphone app with personalized information, exercises and physical activity modules is an integral part of physiotherapy treatment. If patients are considered not to be suitable for blended care, a paper-based workbook with a similar content will be integrated with physiotherapy treatment. A more detailed description of both modes of care delivery is provided below. The content and intensity of the physiotherapy treatment was based on the Dutch KNGF Clinical Practice Guidelines for Physiotherapy Neck pain, complaints of the arms, neck and/or shoulder (CANS) and Subacromial Complaints [9–11]. An overview of the Stratified Blended Approach is Provided in Table 1.

**Table 1:** Overview of the Stratified Blended Approach

**Phase 1: Stratification**			
**Content and intensity of treatment** will be matched to the patient’s risk of persistent disabling pain (low, medium or high, assessed with the Keele STarT MSK Tool)		**Mode of care delivery** will be matched to the patient’s suitability for blended care (yes or no, assessed with the Dutch Blended Physiotherapy Checklist)	
**Phase 2: Matched treatment per risk profile**			
	**Low risk**	**Medium risk**	**High risk**
*Physiotherapy sessions (either face-to-face or video consults)*			
**Aim**	Improvement of a patient’s pain and disability		
**Intensity**	3–4 sessions (3 weeks)	6–9 sessions (12 weeks)	8–12 sessions (12 weeks)
**Content**	Reassurance, provide information on neck/shoulder complaints, personal etiology, self-management options and the importance of adequate physical activity/exercise behavior	Similar to low risk and additionally: consider to provide passive or active joint mobilization techniques, in combination with functional exercise therapy	Similar to medium risk and additionally: consider to address patient’s specific physical and psychosocial obstacles to recovery, using a combination of physical and psychological approaches, including pain education
**Integration**	Motivate to read information modules and do home-based exercises independently	Per session evaluation of progress with e-Exercise app or paper-based workbook to optimize physiotherapy treatment	
**Evaluation**	A final session to evaluate the progress and give recommendations to prevent recurrent episodes of neck/shoulder complaints and maintain or improve the physical activity level		
*Patient’s home setting: e-Exercise app or paper-based workbook*			
**Information module**	3 weekly varying information themes, including assignments to stimulate self-reflection	12 weekly varying information themes, including assignments to stimulate self-reflection, about the etiology of neck/shoulder complaints, physical activity, patient experiences, pain management, and psychosocial factors related to neck/shoulder complaints. The information and order of the information provided will differ per risk profile and working status	
**Exercise module**	3–4 personalized exercises to fit the patient’s specific functional status.		
**Physical activity module**	Recommendations	The patient chooses one physical activity and sets a goal to maintain or enhance the level of that physical activity. A graded activity functionality can be activated.	The patient chooses one physical activity and sets a goal to enhance the level of that physical activity, by using a graded activity functionality.

#### Matched treatment: content and intensity of physiotherapy care

At the start of the first physiotherapy session patients will complete the Keele STarT MSK Tool, for the physiotherapist to help decide what the content and intensity of the physiotherapy treatment should be. The content and intensity of physiotherapy will be matched to the patient’s risk of persistent disabling pain (i.e. low, medium or high risk). In addition, content of the e-Exercise functionalities will also differ per risk profile. A more detailed description about the content and intensity per risk level can be found below.

#### Low risk

Patients classified as being at ‘low risk’ of persistent disabling pain will be offered 3 to 4 physiotherapy sessions, on average. During the first session, the physiotherapist will reassure the patient, provide information about neck and/or shoulder complaints (and the personal etiology), will discuss some self-management options as well as the importance of adequate physical activity behavior. Furthermore, the patient will be instructed on three to four personalized home-based exercises that fit the patients’ functional status. The information module within the e-Exercise module will function as a knowledge-based platform through which the neck and/or shoulder self-management information is directly available for the patient. During the final session, the patient’s progress will be evaluated and recommendations will be given to prevent recurrent episodes of neck and/or shoulder complaints and on how to maintain or improve their physical activity level.

#### Medium risk

Patients classified as being at ‘medium risk’ of persistent disabling pain will be offered 6 to 9 physiotherapy sessions, on average. In addition to the content of the “low risk” protocol, physiotherapists can consider to provide additional evidence-based interventions as recommended by the guidelines of the Royal Dutch Association for Physiotherapy (KNGF). Examples of such exercises are passive or active joint mobilization in combination with functional exercise therapy [9–11]. During each physiotherapy session, the physiotherapist will evaluate the progress of the patient using the e-Exercise app or a paper-based workbook to optimize physiotherapy care. The information module of the e-Exercise module will contain 12 weekly varying self-management themes, including assignments. The physical activity module can be used to maintain or enhance patients’ level of physical activity. Depending on the patients’ pain-related fears for physical activity, the graded activity functionality can be activated.

#### High risk

Patients classified as being at ‘high risk’ of persistent disabling pain will be offered 8 to 12 physiotherapy sessions, on average. In addition to the content of the “medium risk” protocol, the physiotherapist is asked to focus on addressing physical and psychosocial obstacles to recovery by using a combination of physical and psychological approaches, including pain education and graded activity principles. The information module of the e-Exercise module will contain the same 12 themes as the ‘medium risk’ protocol, but the themes focusing on psychosocial obstacles will be addressed in an earlier stage. The physical activity module of e-Exercise and the workbook will be used to enhance the level of physical activity, by using graded activity principles.

#### Matched treatment: mode of care delivery

During the first physiotherapy session, the physiotherapist will use the Dutch Blended Physiotherapy Checklist to decide whether blended care or the integration of a paper-based workbook is the most suitable mode of care delivery of primary care physiotherapy for an individual patient.

#### Blended care

If considered suitable for blended care, the patient will be offered a blended physiotherapy treatment (e-Exercise) in which a smartphone app with e-Exercise modules plays an integral part of physiotherapy treatment. The functionalities of e-Exercise neck, shoulder, and neck and shoulder are integrated within the MijnZorgApp (www.mijnzorgapp.com), which was developed by The Health Train BV. A photo of a person using one of the e-Exercise modules of MijnZorgApp is provided in Appendix 1. The e-Exercise functionalities consist of three integrated modules, which differ per risk profile and will be personalized for each individual patient:

1. An information module, containing various themes (text and video or animation), including assignments to stimulate self-reflection, about the etiology of neck and shoulder complaints, physical activity, patient experiences, pain management, and psychosocial factors related to neck and/or shoulder complaints. Which information is provided to the patient, and the order of the provided information, will differ per risk profile and working status. Reminders will be sent each week;
2. An exercise module, including video-instructed exercises. The physiotherapist chooses exercises that fit the patient’s specific functional status best. The app will send daily push-reminders to the patient to remind them to exercise. These reminders can be adjusted to fit the patient’s schedule. After each exercise session, the patient will evaluate the session in the app. Both patients and physiotherapists will be able to monitor the progress;
3. A physical activity module, containing physical activity recommendations. At the start of the treatment, the patient chooses one physical activity (e.g. walking, running or cycling) and sets a long-term goal for that physical activity. The physical activity module can be used to maintain or enhance the patients’ level of physical activity. If the patient experiences pain-related fears for physical activity, a graded activity functionality can be activated [17, 19, 28]. Within this functionality, physical activity recommendations gradually increase to reach patients’ individual goal within 12 weeks. Patients will be able to self-monitor their progress and will receive tailored feedback about their actual amount of physical activity compared to their recommended amount of physical activity.

Several behavior change techniques are used in the e-Exercise modules: goal setting (behavior and outcome), review behavior goals, feedback on behavior, self-monitoring of behavior, social support, instruction on how to perform the behavior, information about health consequences, information about emotion consequences, demonstration of the behavior, social comparison, prompts/cues, reduce prompts/cues, behavior substitution, habit formation, generalization of target behavior, graded tasks, credible source, social reward, social incentive, reduce negative emotions, restructuring the social environment, framing/reframing and focus on past success [22]. For example, the e-Exercise modules ask patients to plan exercises and physical activities and patients’ can monitor their treatment progress. After concluding physiotherapy treatment, agreements made between patients and physiotherapists are noted and six messages will be sent to remind patients on the lessons learnt (every 14 days). Additionally, the e-Exercise information modules will stay available for patients to review or reread information at any time without an end-date.

#### Paper-based workbook

If a patient is considered more suitable for the integration of a paper-based workbook rather than blended care, a paper-based workbook will be integrated in the physiotherapy treatment. The paper-based workbook consists of similar modules as the e-Exercise app, but without the video’s, animations, reminders, tailored feedback, and content matching on risk profile, type of complaints, and work status.

#### Usual physiotherapy

Patients in the usual physiotherapy arm will be offered usual care (face-to-face or video consults) based upon the recommendations of the guidelines of the Royal Dutch Association for Physiotherapy (KNGF) [9–11]. The clinical guideline for neck pain, recommends categorization in treatment profiles based on: the severity of neck pain, the course of symptoms (normal vs. deviant) and the presence of psychosocial factors that may hinder recovery (yes vs. no). The clinical guideline for complaints of the arm, neck and shoulder recommends categorization in treatment profiles based on the region of complaints indicated as most problematic and the relationship between complaints, disabilities, and limitations in participation. No stratification tools to identify patient subgroups and subsequently match them to a treatment are recommended by the guidelines.

### Measurements

The first assessment will consist of a digital questionnaire and an accelerometer that will be sent to the patients by mail and patients will be asked to wear the accelerometer for five consecutive days. Patients will be asked to complete the first questionnaire within one week after starting physiotherapy treatment. If the first digital questionnaire is not completed within two weeks after starting physiotherapy treatment, clinical data of the first questionnaire will not be included in the data analyses. Outcomes will be measured again at 3, 6, and 9 months after the first digital questionnaire was completed. A schedule of enrolment, interventions, and assessments is provided in Table 2.

**Table 2:**
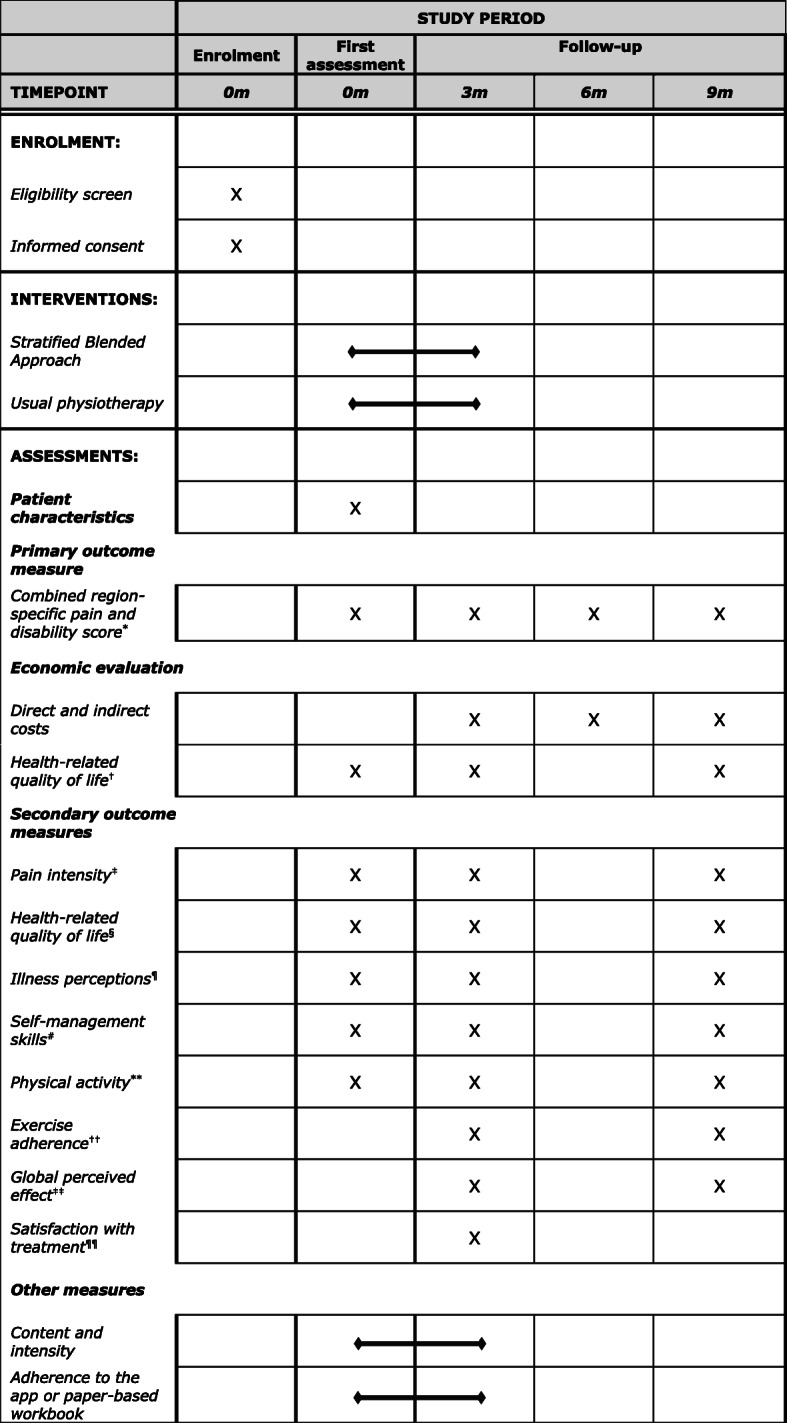
Schedule of enrolment, interventions, and assessments

Indicator for a period; duration of the period is not limited to length of the indicator and dependent on duration of interventions, *: Neck Pain and Disability Scale or Shoulder Pain and Disability Index, ^†^: EuroQol group instrument with 5 levels of severity for each of the 5 dimensions, ^‡^: Numeric Rating Scale Pain, ^§^: 36-Item Short Form Health Survey, ^¶^: Brief Illness Perception Questionnaire, ^#^: Dutch version of the short form Patient Activation Measure, ^**^: ActiGraph accelerometer, ^††^: Exercise Adherence Rating Scale, ^‡‡^: Global Perceived Effect scale, ^¶¶^: 8-point Likert scale

#### Primary outcome

- The primary outcome is the combined region-specific pain and disability score over 9 months follow-up, assessed by the Neck Pain and Disability Scale (NPAD) [29–33] for patients with primarily neck complaints and by the Shoulder Pain and Disability Index (SPADI) [34–38] for patients with primarily shoulder complaints. A higher total score (0–100 for both outcome measures) indicates increased pain and functional limitations [29–38].

#### Secondary outcomes

- The average neck and/or shoulder pain intensity in the last week will be measured with an 11-point Numeric Rating Scale (NRS) (0 = no pain; 10 = worst pain imaginable) [39, 40].
- Health-related quality of life will be measured with the 36-Item Short Form Health Survey (SF-36). The questionnaire consists of eight subscales (physical functioning, role limitations due to physical health, role limitations due to emotional problems, energy/fatigue, emotional well-being, social functioning, pain (over the last 4 weeks) and general health). Scores for each subscale will be calculated (0–100). Higher scores indicate a better health-related quality of life [41–45].
- Illness perceptions will be measured with the Brief Illness Perception Questionnaire (IPQ-K) [46–48]. This questionnaire is an eight-item scale designed to assess cognitive and emotional representations of illness on an ordinal scale (0–10) [46–48].
- Patients’ self-management skills are assessed with the Dutch version of the short form Patient Activation Measure (PAM13-Dutch) [49, 50]. The PAM 13-Dutch is a reliable 13-item instrument and assesses patient (or consumer) self-reported knowledge, skills and confidence for self-management of one’s health or chronic condition. The answering categories per item are 4-point Likert scales, ranging from totally disagree to totally agree and ‘non applicable’. A higher score (range 1–100) indicates a higher level of self-management [49, 50].
- Physical activity will be objectively measured with an Actigraph accelerometer [51, 52]. The Actigraph accelerometer is a reliable tool for measuring physical activity in adults. Participants will be instructed to wear the accelerometer on their waist for five consecutive days, except when sleeping, showering, bathing or swimming [51, 52]. Average amount of moderate or vigorous physical activity (MVPA) per day will be calculated.
- Exercise adherence will be measured with the Exercise Adherence Rating Scale (EARS) [53]. The EARS is a 6 item self-reported questionnaire with items scored on a 5-point Likert scale (0 = completely agree; 4 = completely disagree). A higher score (0–24) indicates better adherence to prescribed home-exercises [53].
- Global perceived effect will be measured with the 7-point Likert global perceived effect score (GPE) [54, 55]. Categories 1 (very much improved) to 3 (a little improved) are classified as ‘improved’. Categories 4 (no change) to 7 (very much worse) are classified as ‘not improved’ [54, 55].
- Satisfaction with treatment outcome will be measured with an 8-point Likert scale question: ‘All things considered, how satisfied are you with the results of the treatment for your neck and/or shoulder complaints? (1 = extremely satisfied, 7 = extremely dissatisfied, 8 = not sure/no opinion) [56].

### Demographic and clinical characteristics

- Patient characteristics are only collected in the first questionnaire and include various demographic and clinical variables, including: age, sex, education level, duration of complaints, weight, height and co-morbidities.
- The risk of persistent disabling pain will be assessed with the Keele STarT MSK Tool (i.e. low, medium or high risk) [13]. The Keele STarT MSK Tool is part of the Stratified Blended Approach and is additionally included in the data collection.
- As part of the data collection, patients’ suitability for e-Exercise (blended care) will be measured by two self-developed questions as substitute for the Dutch Blended Physiotherapy Checklist. It is not possible to use the Dutch Blended Physiotherapy Checklist as a measurement instrument, because it is a tool to guide physiotherapists in their clinical reasoning while setting up a personalized blended physiotherapy treatment, thus not a patient reported outcome measure [16]. Therefore, this cannot be measured in the control arm. The following questions will be assessed in the first questionnaire: ‘Do you own a smartphone or tablet? (yes/no)’ and ‘How many apps do you use regularly (weekly) on your smartphone or tablet? (none/1-3 per week/4-10 per week/more than 10 per week)’.

### Other outcomes

- The content and intensity of physiotherapy care will be measured by a case report form, filled out by the physiotherapist at the end of the treatment period or after 3 months. Information of the risk of persistent disabling pain, the suitability for blended care, the physiotherapists diagnosis of the presenting problem, the number of physiotherapy sessions, deviations from the study protocol, and content of the physiotherapy sessions will be collected.
- Adherence to the smartphone app with e-Exercise modules in the Stratified Blended Approach arm will be assessed by quantitative data on the usage. These data will automatically be stored on the backend of the app. Additionally, all patients will be asked in the first follow-up questionnaire whether they received and used an app or paper-based workbook as part of their physiotherapy treatment.

### Sample size

The sample size calculation is based on the primary outcome, i.e. the difference in the combined pain and disability score over 9 months between Stratified Blended Approach and usual physiotherapy. The sample size is based on the following assumptions: an intracluster correlation coefficient (ICC) of 0.04, 92 clusters in the analyses (individual physiotherapists), an average cluster size of 3, an expected between arm difference in effectiveness of > 10 out of 100 in half of the study population, a power of 80%, and an alpha of 0.05 [57]. An ICC of 0.04 was used, because of the expected clustering effect in the outcomes of patients being treated by the same physiotherapist [58–60]. ICCs smaller than 0.05 are typical for patient-reported outcomes in cluster randomized trials [57]. With an ICC of 0.04, and a cluster size of 3, the number of physiotherapists required to achieve the adequate statistical power is 92. We assume a minimal clinical important difference of > 10 points on the Neck Pain And Disability Scale and Shoulder Pain And Disability Index, but expect superiority of the Stratified Blended Approach over usual care in only half of the trial population (i.e. those patients at medium and high risk of persistent disabling pain), and a standard deviation of 20 [29, 30, 34]. That would lead to an overall effect size of 0.25 between the two arms. We assume no clinically important between arm difference in patients at low risk of persistent disabling pain, given they are expected to have a good prognosis irrespective of treatment. After the first measurement, we will perform three follow-up measures. Having three repeated measures decreases the required sample size by 27% [61]. Based on these assumptions, a total sample size of 202 patients is needed. After correcting for an expected loss to follow-up rate of 15% over 9 months follow-up, a total of 238 patients (119 per arm) is needed.

### Statistical analysis

Descriptive statistics (e.g. means and proportions) will be used to describe the main characteristics of the clusters (physiotherapists) and trial population (patients). Main characteristics of physiotherapists that will be reported are: sex, age, specialization, years of experience working as a physiotherapist, employment status and physiotherapy practice size (where the physiotherapist is employed). The demographic and clinical variables of patients collected in the first questionnaire will be compared (frequencies, t-test, Chi-square) to investigate potential selection bias. Demographic and clinical baseline measurements of dropouts and non-dropouts will be compared to investigate selective attrition. All analyses will be performed according to the ‘intention-to-treat’ principle. Any missing values will be imputed using ‘Multivariate Imputation by Chained Equations’, under the assumption that data are missing at random [62]. Additionally, per protocol analyses will be carried out with people that adhered to the paper-based workbook or the e-Exercise modules. Participants will be considered adherent to the e-Exercise modules if they log in once a week in 67% (low risk) or 75% (medium/high risk) of the total amount of weeks (low risk: 2 weeks over 3 weeks; medium/high risk: 9 weeks over 12 weeks). Participants will be considered to adhere to the paper-based workbook if they self-report that they used the workbook at T1 (3 months). For all analyses, a two-tailed significance level of *p* < 0.05 is considered to be statistically significant. Statistical analysis will be performed using IBM SPSS or statistical package STATA. During the analyses, the researchers will be blinded to group allocation until the entire analysis will be completed.

#### Effectiveness

To determine the overall effectiveness of the Stratified Blended Approach on the combined pain and disability score compared to usual physiotherapy in neck/shoulder patients over 9 months, differences in change scores per arm and time period will be estimated using linear mixed models (LMM) with random effects to control for correlation within patients and physiotherapists. Three levels are identified, consisting of repeated measurements (level 1), nested within patients (level 2), nested within physiotherapists (level 3). Analyses will be controlled for the values at the first measurement and possible confounders, e.g. age, sex, type of complaints (neck or shoulder), pain intensity, duration of complaints [63–68].

The statistical analysis of the primary outcome will also be used for the secondary outcomes. However, for dichotomous outcomes, a generalized mixed model (logit link) with the same multilevel structure will be used. Exploratory subgroup analyses will be carried out for hypotheses generating purposes. These analyses will be carried out to investigate potential differences in effectiveness within the three prognostic risk groups (low, medium or high risk), groups based on suitability for blended care (yes or no) and the neck and shoulder patient groups (self-reported dominantly apparent neck complaints or shoulder complaints).

#### Economic evaluation

A cost-utility analysis (CUA) will be performed for QALYs and a cost-effectiveness analysis (CEA) for the combined region-specific pain and disability score, both of which will be performed from the societal and the healthcare perspective. From the societal perspective all costs will be taken into account, irrespective of who pays or benefits, whereas solely those borne by the healthcare sector will be included if the healthcare perspective is applied [69].

##### Identification, measurement and valuation of costs

Societal costs will be determined during 9 months of follow-up by gathering information on the patients’ healthcare utilization, informal care, and (unpaid) productivity losses due to neck and/or shoulder complaints. This will be done by asking patients to complete three retrospective 3-monthly cost questionnaires. The costs of the Stratified Blended Approach will be estimated using a bottom-up micro costing approach [70]. Other kinds of healthcare utilization will include the use of primary care, secondary care, and medication, all of which will be assessed by the cost questionnaires and valued using Dutch standard costs [69]. If standard costs are unavailable, prices reported by professional organizations will be used. Unpaid productivity losses will be valued using a Dutch recommended shadow price [69]. Paid productivity losses comprise of both sickness absence and presenteeism (i.e. reduced productivity while at work). Sickness absence will be assessed using a modified version of the iMTA Productivity Cost Questionnaire (iPCQ) and will be valued in accordance with the “Friction Cost Approach” (FCA), with a friction period of 12 weeks and gender-specific price weights [69, 71]. The FCA assumes that production losses are confined to the “friction period” (i.e. time needed to replace a sick worker) [71]. The participants’ level of presenteeism will be measured using the “World Health Organization – Work Performance Questionnaire” as well as a modified version of the iPCQ, and will be valued using gender-specific price weights as well [69, 71–73].

##### Measurement and valuation of health-related quality of life

Health-related quality of life will also be measured with the EQ-5D-5L. This questionnaire measures the patients’ severity of complaints on five health domains (i.e. mobility, self-care, usual activities, pain/discomfort and anxiety/depression) [74]. For the cost-utility analysis (CUA), EQ-5D-5L health states will be converted into utility values using the Dutch tariff [75]. Subsequently, Quality Adjusted Life Years (QALYs) will be estimated by multiplying the duration a patient spent in a certain health state by the utility value of that health state, using linear interpolation between measurement points.

##### Statistical analyses

For the CUA and CEA, missing cost and effect data will be imputed using multivariate imputation by chained equations [62]. The results of the imputed datasets will be pooled using Rubin’s rules [62]. LMM, with the same three-level structure as described above, will be performed to estimate cost and effect differences [76]. In order to account for the highly skewed nature of cost data, bias-corrected and accelerated bootstrapping with 5000 replications will be used to estimate 95% confidence intervals around the cost differences. Incremental Cost-Effectiveness Ratios (ICERs) will subsequently be calculated by dividing the differences in costs between study groups by the difference in QALYs for the CUA and the differences in the region-specific pain and disability score for the CEA. The uncertainty surrounding the ICERs will be graphically illustrated by plotting bootstrapped incremental cost-effect pairs on cost-effectiveness planes [77]. Moreover, cost-effectiveness acceptability curves (CEACs) will be constructed to provide a summary measure of the joint uncertainty of costs and effects. CEACs indicate the probability of the intervention being cost-effective in comparison to usual care at different willingness-to-pay values [78]. To test the robustness of the study results, several sensitivity analyses will be performed.

## Discussion

This paper describes the design and methods of a multicenter, pragmatic, two-arm, parallel-group, cluster randomized controlled trial on the effectiveness and cost-effectiveness of the so-called Stratified Blended Approach for people with neck and/or shoulder complaints, compared to usual primary physiotherapy care. Physiotherapy has shown to be effective in reducing pain and disability in patients with neck and/or shoulder complaints [6–11]. However, like other musculoskeletal conditions, there is no ‘one size fits all’ strategy to manage patients with neck and/or shoulder complaints [6]. Subgroups of patients can be identified who are at low, medium or high risk of persistent disabling pain [13, 14] and who are or are not suitable for the integration of a digital application with physiotherapy treatment, called blended care [16]. Identification of these subgroups can help to match the patient to the most appropriate content and intensity of physiotherapy, as well as the most appropriate mode of care delivery. Integrating knowledge from both prognostic risk stratification and blended care might improve the clinical effectiveness and cost-effectiveness of physiotherapy care for patients with neck and shoulder complaints.

Although the trial is well-planned, there will be several operational challenges. The first challenge will be the active participation of physiotherapy practices in the recruitment of patients with neck and/or shoulder complaints to achieve the desired statistical power. During the previous feasibility study (van Tilburg M, Kloek CJJ, Pisters MF, Staal JB, Ostelo WJG, Foster NE, et al: Development & feasibility of a stratified approach integrated with eHealth in patients with neck and/or shoulder complaints, in preparation) we noticed that recruitment of patients was slower than expected. The reasons for this were not related to the interventions, but various procedural and environmental barriers were reported by the participating physiotherapists, that can be overcome in this trial. Because recruitment will predominately be done physiotherapy practice staff, they will be involved and receive clear instructions from the researchers. Furthermore, all physiotherapists will be sent weekly updates by email on the trial progress and latest news and a researcher will have phone contact with poorly recruiting physiotherapy practices to try to address recruitment barriers. Additionally, the COVID-19 crisis in 2020 is another barrier for the recruitment of patients. We already had to a delay the start of the trial and various contact restrictions might lead to a slower recruitment of patients.

A second challenge will be the change in existing work routines of physiotherapists. A previous study showed that implementing a blended intervention into daily routine is a complex process [17]. Since the blended intervention is only one of the components of the Stratified Blended Approach, an extensive training will be essential to ensure that physiotherapists will work according the Stratified Blended Approach as planned. Physiotherapists will receive two training sessions on how to work according to the Stratified Blended Approach, both of which will last half a day. During and in-between these training sessions, physiotherapists will gain experience with working according to the Stratified Blended Approach. Besides the training, physiotherapists will be supported with an informative factsheet containing a summary of the Stratified Blended Approach, a copy of the full written protocol and they will be contacted weekly to ask whether they have questions regarding the Stratified Blended Approach. Additionally, since the COVID-19 crisis in 2020, physiotherapists have been predominately working remotely. We therefore expect physiotherapists to be more open to blended care.

The design of this trial has several strengths. The first strength is the pragmatic design of the trial. Traditional exploratory trials test whether an intervention is beneficial in an ideal situation, whereas pragmatic trials assess the effect of offering the intervention in real clinical practice, increasing the external validity of the results [79, 80]. Pragmatic trials work especially well for complex interventions, such as the Stratified Blended Approach [79, 80]. The broad inclusion criteria for physiotherapists and patients, the relatively high level of flexibility to personalize the components of the Stratified Blended Approach opposed to a strict protocol-based intervention, the range of outcome measures which are directly relevant to participants and comparison to usual physiotherapy will lead to evidence about the real-world effectiveness of this new Stratified Blended Approach [80].

Another strength is the use of cluster randomization at the level of the physiotherapy practice. This design ensures that each participating physiotherapist within a physiotherapy practice delivers either the Stratified Blended Approach or usual physiotherapy, thereby avoiding the risk of contamination [81]. However, due to the lack of blinding of physiotherapists, cluster randomization might lead to selection bias [81]. However, since recruitment of patients in the trial will predominately be done by the other physiotherapy practice staff after first patient contact and not directly by the participating physiotherapists, we hope to minimize any selection bias. To check whether problematic selection bias has occurred after all, demographic and other characteristics of participants will be compared between the intervention and control arm. Cluster randomized trials also require more patients to achieve sufficient power and require more complex analyses [57]. However, in the sample size calculation and statistical analyses, these design effects have been and will be taken into account.

This cluster randomized controlled trial is the first to investigate the clinical effectiveness and cost-effectiveness of a stratified approach integrated with eHealth for people with neck and/or shoulder complaints, compared to usual physiotherapy care. Therefore, it will provide clinically relevant results regarding effectiveness of the Stratified Blended Approach compared to usual care on patients’ pain and disability. These results will help to understand whether integrating stratified care with eHealth in physiotherapy care can improve outcomes for patients.

## Figures and Tables

**Fig. 2:**
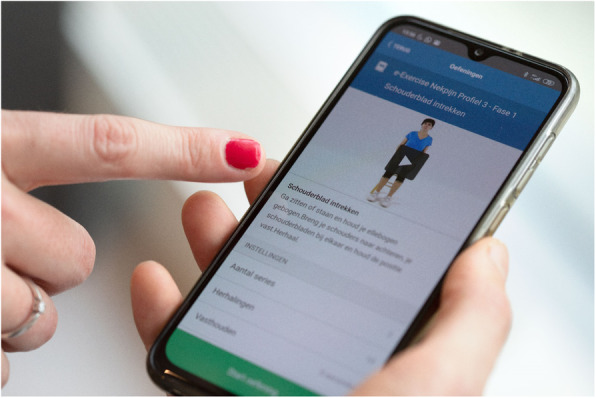
Photo of a person using one of the e-Exercise modules of MijnZorgApp

## Data Availability

Not applicable as this is only a trial protocol.

## Abbreviations

MSK: Musculoskeletal
Keele STarT MSK Tool: Keele Subgroup Targeted Treatment MSK Tool
cRCT: Cluster randomized controlled trial
CeHRes: Center for eHealth Research
CANS: Complaints of the arms, neck and/or shoulder
KNGF: Royal Dutch Association for Physiotherapy (Dutch abbreviation)
NPAD: Neck Pain and Disability Scale
SPADI: Shoulder Pain and Disability Index
NRS: Numeric Rating Scale
SF-36: 36-Item Short Form Health Survey
IPQ-K: Brief Illness Perception Questionnaire
PAM13-Dutch: Dutch version of the short form Patient Activation Measure
MVPA: Moderate or vigorous physical activity
EARS: Exercise Adherence Rating Scale
GPE: Global Perceived Effect
CUA: Cost-utility analysis
CEA: Cost-effectiveness analysis
iPCQ: iMTA Productivity Cost Questionnaire
FCA: Friction Cost Approach
ICERs: Cost-Effectiveness Ratios
CEACs: Cost-effectiveness acceptability curves

## References

[1] James SL, Abate D, Abate KH, Abay SM, Abbafati C, Abbasi N, et al. Global, regional, and national incidence, prevalence, and years lived with disability for 354 diseases and injuries for 195 countries and territories, 1990–2017: a systematic analysis for the Global Burden of Disease Study 2017. Lancet. 2018; 392(10159):1789–858. 10.1016/S0140-6736(18)32279-7 PMC6227754 30496104

[2] Luime JJ, Koes BW, Hendriksen IJM, Burdorf A, Verhagen AP, Miedema HS, et al. Prevalence and incidence of shoulder pain in the general population; a systematic review. Scand J Rheumatol. 2004;33:73–81. 10.1080/03009740310004667 15163107

[3] Parsons S, Breen A, Foster NE, Letley L, Pincus T, Vogel S, et al. Prevalence and comparative troublesomeness by age of musculoskeletal pain in different body locations. Fam Pract. 2007;24(4):308–16. 10.1093/fampra/cmm027 17602173

[4] Hoy DG, Protani M, De R, Buchbinder R. The epidemiology of neck pain. Best Practice and Research: Clinical Rheumatology. 2010;24(6):783–92. 10.1016/j.berh.2011.01.019 21665126

[5] March L, Smith EUR, Hoy DG, Cross MJ, Sanchez-Riera L, Blyth F, et al. Burden of disability due to musculoskeletal (MSK) disorders. Vol. 28, Best Practice and Research: Clinical Rheumatology. 2014. p. 353–66. 10.1016/j.berh.2014.08.002 25481420

[6] Lin I, Wiles L, Waller R, Goucke R, Nagree Y, Gibberd M, et al. What does best practice care for musculoskeletal pain look like? Eleven consistent recommendations from high-quality clinical practice guidelines: Systematic review. Vol. 54, Br J Sports Med; 2020. p. 79–86. 10.1136/bjsports-2018-099878 30826805

[7] Beumer A, de Bie R, ten Cate A, Elders L, van Eijsden-Besseling M, Feleus A, et al. Multidisciplinaire richtlijn aspecifieke Klachten Arm, Nek en/of Schouders [Multidisciplinary guideline non-specific complaints of arm, neck and/or shoulders] (Dutch). Amersfoort: Koninklijk Nederlands Genootschap voor Fysiotherapie (KNGF); 2012.

[8] Côté P, Wong JJ, Sutton D, Shearer HM, Mior S, Randhawa K (2016). Management of neck pain and associated disorders: a clinical practice guideline from the Ontario protocol for traffic injury management (OPTIMa) collaboration. Eur Spine J.

[9] Bier JD, Scholten-Peeters GGM, Staal JB, Pool J, Tulder M van, Beekman E, et al. KNGF-richtlijn Nekpijn. K Ned Genoot voor Fysiother. 2016;V–09.

[10] Heemskerk MAMB, Staal JB, Bierma-Zeinstra SMA, de Haan G, Hagenaars LHA, Lanser K, et al. KNGF-richtlijn Klachten aan de arm, nek en/of schouder (KANS). K Ned Genoot voor Fysiother. 2010:1.

[11] Jansen MJ, Brooijmans F, Geraets JJXR (2011). KNGF evidence statement Subacrominale schouderklachten. Koninglijk Ned Genoot voor Fysiother.

[12] Hingorani AD, Van Der Windt DA, Riley RD, Abrams K, Moons KGM, Steyerberg EW (2013). Prognosis research strategy (PROGRESS) 4: stratified medicine research. BMJ..

[13] Hill JC, Afolabi EK, Lewis M, Dunn KM, Roddy E, Van Der Windt DA (2016). Does a modified STarT Back tool predict outcome with a broader group of musculoskeletal patients than back pain? A secondary analysis of cohort data. BMJ Open.

[14] Hill JC, Garvin S, Chen Y, Cooper V, Wathall S, Saunders B, et al. Stratified primary care versus non-stratified care for musculoskeletal pain: findings from the STarT MSK feasibility and pilot cluster randomized controlled trial. BMC Fam Pract. 2020;21:30. 10.1186/s12875-019-1074-9 PMC7014664 32046647

[15] Campbell P, Hill JC, Protheroe J, Afolabi EK, Lewis M, Beardmore R (2016). Keele aches and pains study protocol: validity, acceptability, and feasibility of the keele start msk tool for subgrouping musculoskeletal patients in primary care. J Pain Res.

[16] Kloek CJJ, Janssen J, Veenhof C (2020). Development of a checklist to assist physiotherapists in determination of patients’ suitability for a blended treatment. Telemed J E Health.

[17] Kloek CJJ, Bossen D, Spreeuwenberg PM, Dekker J, de Bakker DH, Veenhof C. Effectiveness of a blended physical therapist intervention in people with hip osteoarthritis, knee osteoarthritis, or both: a cluster-randomized controlled trial. Phys Ther. 2018;98(7):560–70. 10.1093/ptj/pzy045 PMC6016690 29788253

[18] Kloek CJ, Bossen D, Veenhof C, Van Dongen JM, Dekker J, De Bakker DH. Effectiveness and cost-effectiveness of a blended exercise intervention for patients with hip and/or knee osteoarthritis: Study protocol of a randomized controlled trial. BMC Musculoskelet Disord. 2014;15:269. 10.1186/1471-2474-15-269 PMC4243525 25103686

[19] Kloek CJJ, van Tilburg ML, Staal JB, Veenhof C, Bossen D. Development and proof of concept of a blended physiotherapeutic intervention for patients with non-specific low back pain. Physiotherapy. 2019;105(4):483–91. 10.1016/j.physio.2018.12.006 31031023

[20] Bossen D, Kloek C, Snippe HW, Dekker J, de Bakker D, Veenhof C (2016). A blended intervention for patients with knee and hip osteoarthritis in the physical therapy practice: development and a pilot study. JMIR Res Protoc.

[21] Kloek CJJ, Bossen D, de Vries HJ, de Bakker DH, Veenhof C, Dekker J (2018). Physiotherapists’ experiences with a blended osteoarthritis intervention: a mixed methods study. Physiother Theory Pract.

[22] Michie S, Richardson MS, Johnston M, Abraham C, Francis J, Hardeman W (2013). The behavior change technique taxonomy (v1) of 93 hierarchically clustered techniques: building an international consensus for the reporting of behavior change interventions. Ann Behav Med.

[23] de Vries HJ, Kloek CJJ, de Bakker DH, Dekker J, Bossen D, Veenhof C. Determinants of adherence to the online component of a blended intervention for patients with hip and/or knee osteoarthritis: a mixed methods study embedded in the e-exercise trial. Telemed e-Health. 2017;23(12):1002–10. 10.1089/tmj.2016.0264 28525310

[24] Hill JC, Whitehurst DGT, Lewis M, Bryan S, Dunn KM, Foster NE (2011). Comparison of stratified primary care management for low Back pain with current best practice (STarT Back): a randomised controlled trial. Lancet..

[25] Foster NE, Hill JC, Doyle C, Young J. Effect of Stratified Care for Low Back Pain in Family Practice. Ann Fam Med. 2014;12(2):102–11. 10.1370/afm.1625 PMC3948756 24615305

[26] Huisstede BMA, Miedema HS, Verhagen AP, Koes BW, Verhaar JAN. Multidisciplinary consensus on the terminology and classification of complaints of the arm, neck and/or shoulder. Occup Environ Med. 2007;64(5):313–9. 10.1136/oem.2005.023861 PMC2092547 17043078

[27] van Gemert-Pijnen JEWC, Nijland N, van Limburg M, Ossebaard HC, Kelders SM, Eysenbach G, et al. A holistic framework to improve the uptake and impact of eHealth technologies. J Med Internet Res. 2011;13(4):e111. 10.2196/jmir.1672 PMC3278097 22155738

[28] Bossen D, Veenhof C, Van Beek KE, Spreeuwenberg PM, Dekker J, De Bakker DH. Effectiveness of a web-based physical activity intervention in patients with knee and/or hip osteoarthritis: randomized controlled trial. J Med Internet Res. 2013;15(11):e257. 10.2196/jmir.2662 PMC3841352 24269911

[29] Jorritsma W, Dijkstra PU, De Vries GE, Geertzen JHB, Reneman MF. Detecting relevant changes and responsiveness of Neck Pain and Disability Scale and Neck Disability Index. Eur Spine J. 2012;1(12):2550–7. 10.1007/s00586-012-2407-8 PMC3508212 22752592

[30] Yao M, Xu B Ping, Tian Z Rui, Ye J, Zhang Y, Wang Y Jun, et al. Cross-cultural adaptation of the Neck Pain and Disability Scale: a methodological systematic review. Spine Journal. Elsevier; 2019;19(6):1057–66. 10.1016/j.spinee.2019.01.007 30708113

[31] Jorritsma W, De Vries GE, Dijkstra PU, Geertzen JHB, Reneman MF (2012). Neck pain and disability scale and neck disability index: validity of Dutch language versions. Eur Spine J.

[32] Blozik E, Himmel W, Kochen MM, Herrmann-Lingen C, Scherer M (2011). Sensitivity to change of the neck pain and disability scale. Eur Spine J.

[33] Jorritsma W, De Vries GE, Geertzen JHB, Dijkstra PU, Reneman MF (2010). Neck pain and disability scale and the neck disability index: reproducibility of the Dutch language versions. Eur Spine J.

[34] Thoomes-de Graaf M, Scholten-Peeters W, Duijn E, Karel Y, de Vet HCW, Koes B (2017). The responsiveness and interpretability of the shoulder pain and disability index. J Orthop Sport Phys Ther.

[35] MacDermid JC, Solomon P, Prkachin K (2006). The shoulder pain and disability index demonstrates factor, construct and longitudinal validity. BMC Musculoskelet Disord.

[36] Thoomes-de Graaf M, Scholten-Peeters GGM, Schellingerhout JM, Bourne AM, Buchbinder R, Koehorst M, et al. Evaluation of measurement properties of self-administered PROMs aimed at patients with non-specific shoulder pain and “activity limitations”: a systematic review. Vol. 25. Qual Life Res. 2016:2141–60. 10.1007/s11136-016-1277-7 PMC4980404 27039305

[37] Roe Y, Soberg H, Bautz-Holter E, Ostensjo S (2013). A systematic review of measures of shoulder pain and functioning using the international classification of functioning, disability and health (ICF). BMC Musculoskelet Disord.

[38] Schmidt S, Ferrer M, González M, González N, Valderas JM, Alonso J (2014). Evaluation of shoulder-specific patient-reported outcome measures: a systematic and standardized comparison of available evidence. J Shoulder Elb Surg.

[39] Jensen MP, Turner JA, Romano JM, Fisher LD. Comparative reliability and validity of chronic pain intensity measures. Pain. 1999;83(2):157–62. 10.1016/s0304-3959(99)00101-3 10534586

[40] Breivik EK, Björnsson GA, Skovlund E. A comparison of pain rating scales by sampling from clinical trial data. Clin J Pain. 2000;16(1):22–8. 10.1097/00002508-200003000-00005 10741815

[41] Van Der Zee KI, Sanderman R. Het meten van de algemene gezondheidstoestand met de Rand-36: een handleiding. NCG reeks meetinstrumenten. 2012. ISBN 90-72156-60-9.

[42] Zee KI, Sanderman R, Heyink JW, Haes H. Psychometric qualities of the rand 36-item health survey 1.0: a multidimensional measure of general health status. Int J Behav Med. 1996;3(2):104–22. 10.1207/s15327558ijbm0302_2 16250758

[43] Aaronson NK, Muller M, Cohen PDA, Essink-Bot ML, Fekkes M, Sanderman R (1998). Translation, validation, and norming of the Dutch language version of the SF-36 health survey in community and chronic disease populations. J Clin Epidemiol.

[44] Bullinger M, Alonso J, Apolone G, Leplège A, Sullivan M, Wood-Dauphinee S (1998). Translating health status questionnaires and evaluating their quality: the IQOLA project approach. International quality of life assessment. J Clin Epidemiol.

[45] Ware JE, Gandek B, Kosinski M, Aaronson NK, Apolone G, Brazier J (1998). The equivalence of SF-36 summary health scores estimated using standard and country-specific algorithms in 10 countries: results from the IQOLA project. J Clin Epidemiol.

[46] Leysen M, Nijs J, Meeus M, Paul Van, Wilgen C, Struyf F, Vermandel A, et al. Clinimetric properties of illness perception questionnaire revised (IPQ-R) and brief illness perception questionnaire (Brief IPQ) in patients with musculoskeletal disorders: a systematic review. Man Ther. 2015;20(1):10–7. 10.1016/j.math.2014.05.001 25435470

[47] Hallegraeff JM, Van Der Schans CP, Krijnen WP, De Greef MHG. Measurement of acute nonspecific low back pain perception in primary care physical therapy: reliability and validity of the brief illness perception questionnaire. BMC Musculoskelet Disord. 2013;14:53. 10.1186/1471-2474-14-53 PMC3570488 23369321

[48] Broadbent E, Wilkes C, Koschwanez H, Weinman J, Norton S, Petrie KJ. A systematic review and meta-analysis of the Brief Illness Perception Questionnaire. Psychol Heal. 2015;30(11):1361–85. 10.1080/08870446.2015.1070851 26181764

[49] Hibbard JH, Mahoney ER, Stockard J, Tusler M. Development and testing of a short form of the patient activation measure. Health Serv Res. 2005;40(6 Pt 1):1918–30. 10.1111/j.1475-6773.2005.00438.x PMC1361231 16336556

[50] Rademakers J, Nijman J, Van Der Hoek L, Heijmans M, Rijken M. Measuring patient activation in the Netherlands: translation and validation of the American short form patient activation measure (PAM13). BMC Public Health. 2012;12:577. 10.1186/1471-2458-12-577 PMC3490810 22849664

[51] Robusto KM, Trost SG (2012). Comparison of three generations of ActiGraph™ activity monitors in children and adolescents. J Sports Sci.

[52] Aadland E, Ylvisåker E (2015). Reliability of the actigraph GT3X+ accelerometer in adults under free-living conditions. PLoS One.

[53] Newman-Beinart NA, Norton S, Dowling D, Gavriloff D, Vari C, Weinman JA, et al. The development and initial psychometric evaluation of a measure assessing adherence to prescribed exercise: the Exercise Adherence Rating Scale (EARS). Physiother (United Kingdom). 2017;103(2):180–5. 10.1016/j.physio.2016.11.001 27913064

[54] Kamper SJ, Ostelo RWJG, Knol DL, Maher CG, de Vet HCW, Hancock MJ. Global Perceived Effect scales provided reliable assessments of health transition in people with musculoskeletal disorders, but ratings are strongly influenced by current status. J Clin Epidemiol. 2010; 63(7):760–766.e1. 10.1016/j.jclinepi.2009.09.009 20056385

[55] Evans R, Bronfort G, Maiers M, Schulz C, Hartvigsen J (2014). “I know it’s changed”: a mixed-methods study of the meaning of global perceived effect in chronic neck pain patients. Eur Spine J.

[56] Hudak PL, Wright JG. The characteristics of patient satisfaction measures. Spine. 2000;25(24):3167–77. 10.1097/00007632-200012150-00012 11124733

[57] Campbell MK, Piaggio G, Elbourne DR, Altman DG (2012). Consort 2010 statement: Extension to cluster randomised trials. BMJ.

[58] Eccles M, Grimshaw J, Steen N, Parkin D, Purves I, McColl E (2000). The design and analysis of a randomized controlled trial to evaluate computerized decision support in primary care: the COGENT study. Fam Pract.

[59] Thomas RE, Grimshaw JM, Mollison J, McClinton S, McIntosh E, Deans H (2003). Cluster randomized trial of a guideline-based open access urological investigation service. Fam Pract.

[60] Kerry SM, Bland JM (1998). The intracluster correlation coefficient in cluster randomisation. BMJ..

[61] Vickers AJ (2003). How many repeated measures in repeated measures designs? Statistical issues for comparative trials. BMC Med Res Methodol.

[62] Campion WM, Rubin DB. Multiple imputation for nonresponse in surveys. J Mark Res. 2006. ISBN: 978-0-471-65574-9.

[63] Artus M, Campbell P, Mallen CD, Dunn KM, Van Der Windt DAW. Generic prognostic factors for musculoskeletal pain in primary care: a systematic review. BMJ Open. 2017;7(1):e012901. 10.1136/bmjopen-2016-012901 PMC5253570 28096253

[64] Verwoerd M, Wittink H, Maissan F, de Raaij E, Smeets RJEM (2019). Prognostic factors for persistent pain after a first episode of nonspecific idiopathic, non-traumatic neck pain: a systematic review. Musculoskelet Sci Pract.

[65] Kuijpers T, Van Der Windt DAWM, Van Der Heijden GJMG, Bouter LM (2004). Systematic review of prognostic cohort studies on shoulder disorders. Pain..

[66] Struyf F, Geraets J, Noten S, Meeus M, Nijs J. A multivariable prediction model for the chronification of non-traumatic shoulder pain: A systematic review. Vol. 19, Pain Physician. Am Soc Interv Pain Phys. 2016:1–10. 26815244

[67] Bruls VEJ, Bastiaenen CHG, De Bie RA. Prognostic factors of complaints of arm, neck, and/or shoulder: A systematic review of prospective cohort studies. Vol. 156, Pain. Lippincott Williams and Wilkins. 2015:765–88. 10.1097/j.pain.0000000000000117 25659066

[68] Paksaichol A, Janwantanakul P, Purepong N, Pensri P, Van Der Beek AJ. Office workers’ risk factors for the development of non-specific neck pain: A systematic review of prospective cohort studies. Vol. 69, Occupational and Environmental Medicine. Occup Environ Med. 2012:610–8. 10.1136/oemed-2011-100459 22581966

[69] Hakkaart-van Roijen L, Van der Linden N, Bouwmans CAM, Kanters TA, Tan SS. Costing manual: methodology of costing research and reference prices for economic evaluations in healthcare [in Dutch: Kostenhandleiding: Methodologie van kostenonderzoek en referentieprijzen voor economische evaluaties in de gezondheidszorg]. 2015.

[70] Frick KD. Microcosting quantity data collection methods. Med Care. 2009;47(7 Suppl 1):S76–81. 10.1097/MLR.0b013e31819bc064 PMC2714580 19536026

[71] Koopmanschap MA, Rutten FFH, van Ineveld BM, van Roijen L. The friction cost method for measuring indirect costs of disease. J Health Econ. 1995;14(2):171–89. 10.1016/0167-6296(94)00044-5 10154656

[72] Kessler RC, Barber C, Beck A, Berglund P, Cleary PD, McKenas D, et al. The World Health Organization health and work performance questionnaire (HPQ). J Occup Environ Med. 2003;45(2):156–74. 10.1097/01.jom.0000052967.43131.51 12625231

[73] Kessler RC, Ames M, Hymel PA, Loeppke R, McKenas DK, Richling DE, et al. Using the World Health Organization health and work performance questionnaire (HPQ) to evaluate the indirect workplace costs of illness. J Occup Environ Med. 2004;46(6 Suppl):S23–37. 10.1097/01.jom.0000126683.75201.c5 15194893

[74] EuroQol Group. EuroQol--a new facility for the measurement of health-related quality of life. Health Policy. 1990;16(3):199–208. 10.1016/0168-8510(90)90421-9 10109801

[75] Versteegh M, Vermeulen KM, Evers S MAA, De Wit GA, Prenger R, Stolk EA. Dutch tariff for the five-level version of EQ-5D. Value Heal 2016;19(4):343–352. 10.1016/j.jval.2016.01.003 27325326

[76] El Alili M, van Dongen JM, Goldfeld KS, Heymans MW, van Tulder MW, Bosmans JE. Taking the analysis of trial-based economic evaluations to the next level: the importance of accounting for clustering. Pharmacoeconomics. 2020:30. 10.1007/s40273-020-00946-y PMC7546992 32729091

[77] Black WC. The CE Plane: a graphic representation of cost- effectiveness. Med Decis Making. 1990;10:212–4. 10.1177/0272989X9001000308 2115096

[78] Fenwick E, O’Brien BJ, Briggs A. Cost-effectiveness acceptability curves - facts, fallacies and frequently asked questions. Health Econ. 2004;13(5):405–15. 10.1002/hec.903 15127421

[79] Godwin M, Ruhland L, Casson I, MacDonald S, Delva D, Birtwhistle R (2003). Pragmatic controlled clinical trials in primary care: the struggle between external and internal validity. BMC Med Res Methodol.

[80] Ford I, Norrie J (2016). Pragmatic trials. Drazen JM, Harrington DP, McMurray JJV, Ware JH, woodcock J, editors. N Engl J Med.

[81] Lorenz E, Köpke S, Pfaff H. Blettner M. Cluster-randomized studies - Part 25 of a series on evaluating scientific publications. Vol. 115, Deutsches Arzteblatt International. Deutscher Arzte-Verlag GmbH. 2018:163–8. 10.3238/arztebl.2018.0163 PMC5881078 29587960

